# Psychosocial distress, clinical variables and self-management activities associated with type 2 diabetes: a study in Ghana

**DOI:** 10.1186/s40842-020-00102-7

**Published:** 2020-07-14

**Authors:** Margaret Amankwah-Poku, Albert G. B. Amoah, Araba Sefa-Dedeh, Josephine Akpalu

**Affiliations:** 1 Department of Psychology, School of Social Sciences, College of Humanities, University of Ghana, P.O. Box L 84, Legon, Accra, Ghana; 2 Department of Medicine and Therapeutics, University of Ghana Medical School, College of Health Sciences, University of Ghana, Legon, Accra, Ghana; 3 Department of Psychiatry, University of Ghana Medical School, College of Health Sciences, University of Ghana, Legon, Accra, Ghana

**Keywords:** Type 2 diabetes, Psychosocial care, Diabetes distress, Depression, family support, Glycaemic control

## Abstract

**Aim:**

Psychosocial distress can act as a barrier to diabetes self-care management and thus compromise diabetes control. Yet in Ghana, healthcare centres mainly focus on the medical aspect of diabetes to the neglect of psychosocial care. This study determined the relationship amongst psychosocial distress, clinical variables, and self-management activities associated with type 2 diabetes management.

**Method:**

Questionnaires were administered to 162 patients from four hospitals in Accra, Ghana, to assess psychosocial distress (e.g. diabetes distress), clinical variables (e.g. glycaemic control), and self- management activities (e.g. medication intake) related to diabetes. In assessing diabetes distress, the use of the PAID allowed evaluation of broader range of emotional concerns (diabetes-related emotional distress), while the DDS allowed evaluation of factors more closely related to diabetes self-management (diabetes distress).

**Results:**

Diabetes-related emotional distress, diabetes distress and depressive symptoms were reciprocally positively correlated, while non-supportive family behaviour correlated negatively with these psychological variables. Diabetes-related emotional distress correlated positively with systolic and diastolic blood pressure, and correlated negatively with exercise regimen. On the other hand, diabetes distress correlated negatively with dietary and exercise regimen and correlated positively with glycaemic levels, while depressive symptoms correlated positively with glycaemic levels, diabetes complication and systolic blood pressure. Contrary to the literature, non-supportive family behaviour correlated positively with diet, exercise and medication regimen.

**Conclusion:**

The positive association of psychological variables with glycaemic levels and blood pressure levels, and the positive association of non-supportive family behaviour with self-management activities suggests the need for psychosocial care to be incorporate in the management of type 2 diabetes in Ghana. Patients can be screened for diabetes-related distress and symptoms of depression and provided psychosocial care where necessary.

## Introduction

Type 2 diabetes mellitus is a major chronic non-communicable disease that is on the increase globally. It has been estimated that 425 million individuals had the condition in 2017 and by 2045 this number will rise to 629 million [1]. Indeed, the sub-Saharan African region which includes Ghana, is expected to have one of the highest rates of diabetes in the world [1]. It has been well established that psychological distress can impede diabetes self-care management because it can act as barrier to care [2, 3]. The barrier when not addressed can contribute to decreased physical and mental quality of life [4] and escalate into psychological disorders, which can hinder self-care behaviour and thus, compromise diabetes control.

Poor glycaemic control has been associated with higher diabetes-related distress and impaired health related quality of life [5, 6]. Patients with diabetes are therefore at increased risk of decreased psychological well-being [7, 8]. Diabetes distress has been positively associated with, and predicted poor glycaemic control among Ghanaians patients [9]. Aikens [10] suggests that while high diabetes-related distress might predict reduced medication adherence and poor glycaemic control, depressive symptoms may be more important to lifestyle-oriented behaviours and blood glucose self-testing.

Social support (from family and healthcare providers) is also very important for maintaining lifestyle changes and effective diabetes self-management [11]. Increase in social support for adults with diabetes has been associated with a decrease in emotional distress, and individuals with high levels of social support have reported better levels of well-being ([12]. Thus, diabetes treatment does not only require medical treatment but psychosocial care to deal with diabetes-related emotional distress, and in some cases depression, which are commonly associated with diabetes [13].

Literature on psychosocial aspect of diabetes care in sub-Saharan Africa is very scanty, thus, knowledge in this area is lacking and there is the need to bridge this gap as psychosocial care is key to good glycaemic control. For instance, Stephani, Opoku and Beran [14] in a systematic review of diabetes self-management in sub-Saharan Africa, identified only three observational studies that reported about the psychosocial aspects of diabetes. Another systematic review to assess factors influencing type 2 diabetes management in Nigeria, only six studies reported psychosocial-related factors as influencing patients’ management [15]. Young-Hyman et al. [16] recommended amongst others the integration of psychosocial care with collaborative, patient-centred medical care, for optimal health outcomes. Unfortunately, healthcare centres in Ghana mainly focus on the medical aspect of diabetes to the neglect of psychosocial care.

The present study therefore sought to investigate psychosocial distress, which is vital in the care and management of diabetes, but may not be the central focus for diabetes healthcare providers in Ghana. The researchers predicted that psychosocial distress (diabetes-related emotional distress, diabetes distress, depressive symptoms and non-supportive family behaviours) will: a) intercorrelate positively with each other, b) correlate positively with diabetes care-related clinical variables (diabetes duration, body mass index, glycaemic control, diabetes complications, other medical conditions and blood pressure) and c) correlate negatively with diabetes self-management activities (diet, exercise, medication). This study is part of a bigger study that investigated psychosocial barriers to diabetes self-management and care pathways for type 2 diabetes in Ghana.

## Materials and methods

### Study design

The study was cross-sectional in design. Individuals who shared similar characteristics were tested at one point in time.

### Study sites

Four health facilities were accessed for this study; the Greater Accra Regional Hospital, La Hospital, Achimota Hospital, (all government hospitals in the Accra Metropolis) and the Pentecost Hospital (a church-based hospital). These health facilities are all located in Accra, the capital of Ghana and care providers have been trained in multidisciplinary diabetes care.

### Participants

Inclusion Criteria: Participants were individuals who had been diagnosed with type 2 diabetes for at least 1 year. This duration provides enough time to adjust to the condition and the changes that are associated with it [17].

Exclusion criteria: participants were excluded from the study if they had: a) recent (< 6 months) change in their treatment of diabetes (e.g., switch to insulin, additional injection of insulin), b) co-existing major co-morbidities (e.g. chronic pain, end-stage renal disease) and c) emotional problems or have had a traumatic experience in the past 6 months (e.g. death of a loved one, tragic accidents, diagnosis of a terminal illness etc.).

Power analysis using G*Power calculator [18] determined the sample size for this study. Using Cohen and Cohen’s [19] guidelines for power analysis, given an alpha level of 0.05, a power level of .80 with a medium effect size of 0.3, a sample size of 84 was estimated to be adequate for testing. Though a minimum of 84 participants were required for the study, to allow testing of a larger sample, and also taking into consideration that surveys do not have a 100% response rate, this sample size was increased. Thus, 180 patients were conveniently sampled from the four health facilities, of which 162 patients were included in the study as follows; Pentecost hospital, *n* = 39, La General Hospital, *n* = 46, Achimota hospital, n = 39, and Ridge hospital, *n* = 38. Of the 18 participants excluded, four had changed their medication but did not disclose it until after testing, while the rest did not have their HbAlc measured or did not complete the questionnaires.

### Psychosocial variables investigated

To assess psychosocial barriers to wellbeing and quality of life, the following variables were measured, Diabetes distress, Diabetes-related emotional distress, Depression and Non-supportive family behaviour. Diabetes distress and diabetes-related emotional distress are sometimes used interchangeably. However, in this study, these two variables differed in the type of distress they assessed as described below.

#### Diabetes distress

This variable comprised problems and difficulties directly related to diabetes self-management. It represented participants’ distress with diabetes related to the burden of self-management, quality of care from physicians and perceived lack of social support from family and friends. Thus, distress assessed related to different aspects of self-management.

#### Diabetes-related emotional distress

This variable comprised emotional distress and covered concerns related to having diabetes. The measure of this variable covered a broader range of emotional concerns than the diabetes distress variable.

#### Depression

This variable comprised symptoms participants may have experienced related to, mood, pessimism, sense of failure, self-dissatisfaction, guilt, punishment, suicidal ideas, irritability, social withdrawal, indecisiveness, insomnia, loss of appetite, weight loss. The measure of these symptoms determined the presence of depression as well as the intensity of depression.

#### Non-supportive family behaviour

This variable comprised the negative or non-supportive family behaviours that may have influenced participants’ adherence to their diabetes treatment regimen. Such behaviours included criticisms, naggings and arguments with participants about their treatment regimen and glycaemic levels. The frequency of these negative behaviours were assessed.

### Measures

The following measures were administered to participants, all of which were pilot tested for comprehensibility, and as indicated below, the Diabetes Family Behaviour Checklist (DFBC) and the Summary of Diabetes Self−Care Activity Scale (DSCA) scales were modified.

#### Demographic and clinical information

Demographic information obtained included age, sex, occupation, marital status, age of diabetes onset, diabetes treatment, diabetes complications and other medical conditions. Blood pressure measures, height and weight to calculate BMI, and glycated haemoglobin (HbA1c) level (as a measure of glycaemic control) were also obtained, as well as participants’ self-report of whether they maintained healthy eating.

#### Diabetes-related emotional distress

This was assessed using the Problem Area In Diabetes Questionnaire (PAID [20];). The PAID is a 20-item questionnaire with each item representing a unique area of diabetes-related emotional distress. Using a 5-point scale from 0 (not a problem) to 4 (serious problem) it assesses the degree to which each item is perceived as currently problematic. A total score of the item responses reflects the overall level of diabetes-related emotional distress. This scale has high internal reliability (α = .90 [20];). The present sample recorded a Cronbach alpha of .89.

#### Diabetes distress

The Diabetes Distress Scale (DDS 17 [21];) has 17-items rated on a 6-point scale from 1 (not a problem) to 6 (a very serious problem), on which individuals indicate their degree of distress during the past month. The DDS yields a total score and four sub-scale scores: Interpersonal Distress, Regimen-related Distress, Physician-related Distress, and Emotional Burden. Higher scores indicate greater levels of distress. The present study used the total score. Internal reliability of the total DDS score and the four subscales is high (α = .87 [21];) and was the same for the present study (α = .86).

#### Depression

The Beck Depression Inventory (BDI- II [22];) is a 21-item questionnaire with each item consisting of four statements, indicating different levels of severity of a particular symptom. It requires respondents to choose one statement from each item that best describes the way they have been feeling during the past 2 weeks “including today”. Items score from 0 to 3 and are summed up to yield a single depression score. The lower a score the less depressed a person is. Internal reliability in a type 2 diabetes sample has been reported to be .95 [23] and was .84 in the present sample.

#### Family support

This was assessed using the Non-supportive Family Behaviour subscale of the Diabetes Family Behaviour Checklist (DFBC [24];) after pilot testing recorded a low Cronbach alpha for the Supportive Family Behaviour subscale (α = .54). The Non-Supportive Family Behaviour subscale measured the frequency of non-supportive family behaviours (i.e. negative family behaviours) that may influence adherence to treatment regimen on a 4-point scale ranging from 1 (never) to 4 (several times a week). A Cronbach’s alpha of .78 has been reported by Karlsen, Ofedal and Bru, 2012 [25] and an alpha of .74 in the present sample.

#### Diabetes self-care activities

The Summary of Diabetes Self−Care Activity Scale (DSCA [26];) was used as a measure of diabetes self- management. It assesses four areas of diabetes self−care (diet, exercise, blood glucose monitoring and medication intake) over a retrospective 7 − day period, using a 7-point rating scale for exercise and a 4-point and 5-point scale for the other subscales. Vincent, McEwen and Pasvogel [27] report a moderate inter-item reliability (r = .59–.74). In the present study, the blood glucose monitoring subscale was excluded since none of the participants self-tested their blood glucose, due to testing strips being expensive. The present sample recorded a Cronbach alpha of .75 to .80 for the subscales.

### Ethical considerations

The Ethics Committee for Humanities at the University of Ghana (ECH 064/15–16) and the Ghana Health Services Research and Development (GHS-REC 01/07/16) granted ethics approval for this study. The four hospitals also granted permission for the conduct of the study at their facilities.

### Procedure

Data collection took place during diabetes clinics at the outpatient departments. The first author and three research assistants collected the data. During each section, the first author explained to patients the purpose of the study, indicating the inclusion and exclusion criteria. Prospective participants were screened to ensure they were eligible to participate in the study. They were assured of anonymity, confidentiality, and the freedom to participate or withdraw from the study at any time. All participants provided written informed consent.

Testing began by measuring participants’ height. This measure was then entered into an Omron Body Composition Monitor, which was then used to measure their weight and determine their BMI. Blood pressure (BP) levels were then measured using an Omron BP Monitor. The research assistants then proceeded to administer the questionnaires to participants electronically using a Samsung Tablet A. Participants’ blood samples were also taken by finger prick, to test their glycated haemoglobin (HbA1c) using the PTS A1c + kits, which yields results in 5 min. This test was done concurrently with the administration of questionnaires.

### Data analysis

The Statistical Package for Social Sciences (SPSS) version 21, was used to analyse the data collected. Data was analysed to assess the relationship amongst psychosocial variables, clinical variables, and self- management activities associated with type 2 diabetes management. The Pearson’s Product Moment Correlation Coefficient were used for analysis, with 0.05, 0.01 and 0.001 level of significance accordingly.

## Results

One hundred and six-two people with type 2 diabetes completed questionnaires to assess psychosocial variables, clinical and self- management activities related to diabetes and diabetes care. Participants’ demographic and clinical characteristics are presented below.

### Demographic and clinical characteristics of participants

Demographic and clinical characteristics of participants are presented in Table 1. The mean (SD) age of participants was 61.0 (8.1) years with most of them being females (79%). Most of the participants had obtained up to secondary education (46.3%), but less than half of them were in full time employment (40.7%), and more than half of them were married or cohabiting (56.8%). The mean (SD) diabetes duration of participants was 8.2 (5.3) years with most of them on oral medication, and only a few of them (10.5%) admitting to not maintaining a healthy diet. Participants’ Body Mass Index (BMI) indicated on average, an overweight sample. Although their diabetes was fairly controlled with a mean (SD) HbAlc of 7.9 (2.0)% (63 mmol/mol), most participants reported having diabetes complications. A large number of participants reported being hypertensive (79.6%), though systolic blood pressure was within the pre-hypertensive range 137.3(20.9) mmHg, while diastolic blood pressure was normal, 80.1(10.4) mmHg. All hypertensive participants were on antihypertensive medications. None of the participants had a dual diagnosis of major depression, anxiety, bipolar disorder, or any psychological illness in their medical history and thus no record also of using anti-depressants/anti-anxiety medication, as per their self-report and medical records.

**Table 1:** Demographic and clinical characteristics of participants (*n* = 162)

Variable	Frequency (%)	Mean (SD)
*Age (years)*		61.0(8.1)
*Sex*		
Male	34(21.0)	
Female	128 (79)	
*Employment status*		
Working Full Time	66 (40.7)	
Working Part time	07 (4.3)	
Retired	41 (25.3)	
Not employed	48 (29.6)	
*Level of Education*		
Tertiary	14 (8.6)	
Secondary	75 (46.3)	
Vocational	14 (8.6)	
Primary	44 (27.2)	
None	15 (9.3)	
*Marital Status*		
Never married	1(.6)	
Married/cohabiting	92 (56.8)	
Separated	2 (1.2)	
Divorced	24 (14.8)	
Widowed	43 (26.5)	
*Duration of Diabetes (years)*		*8.2 (5.3)*
*Type of Medication*		
Tablet	149 (92.0)	
Tablet, insulin	11 (6.8)	
Insulin	2 (1.2)	
*Maintaining healthy diet*		
No	17 (10.5)	
Yes	145 (89.5)	
*BMI (kg/m2)*		*29 (5.2)*
*HbA1c (%) & (mmol/mol)*		*7.9 (2.0) (63)*
*Diabetes Complications*		
None	29 (17.9)	
Stroke (Only)	1(.6)	
Cardiovascular disease (Only)	1(.6)	
Neuropathy (Only)	30 (18.5)	
Retinopathy (Only)	20 (12.3)	
Erectile dysfunction (Only)	1(.6)	
Combination of complication	80 (49.5)	
*Other Medical Conditions*		
Yes- Hypertension	129 (79.6)	
Yes (Asthma, Ulcer, etc.)	8 (4.9)	
No	25 (15.4)	
*Systolic BP (mmHg)*		*137.3 (20.9)*
*Diastolic BP (mmHg)*		*80.1 (10.4)*

### Scores on psychosocial measures

Means (SD) of psychosocial variables measured are presented in Table 2.

**Table 2:** Means (SD) of psychosocial variables measured

Variable	Mean	SD
PAID	11.1	12.4
DDS (Overall)	26.8	11.8
BDI	8.9	8.2
DFBC	21.6	3.2

*PAID* Problem Area in Diabetes*, DDS* Diabetes Distress Scale

*BDI* Beck Depression Inventory*, DFBC* Diabetes Family Behaviour Scale

Using the MANOVA, differences in psychosocial measures were compared for the following demographic variables: Educational level, Marital status, Employment status and Hospital site. Results showed no significant difference amongst the different levels of the demographic variables. Detailed results are reported in another manuscript under developed.

### Intercorrelations of psychosocial variables

Correlations performed to determine the relationship amongst the psychosocial variables are presented in Table 3. Amongst the psychosocial variables, diabetes-related emotional distress (PAID) correlated positively with diabetes distress (overall DDS), r_(162)_ = .79, *p* < .001 and depressive symptoms (BDI), r_(162)_ = .53, *p <* .001, while diabetes distress, also correlated positively with depressive symptoms, r_(162)_ = .51, *p <* .001, indicating that these variables increased together. On the contrary, non-supportive family behaviour (DFBC) correlated negatively with diabetes-related emotional distress, r_(162)_ = −.26, *p* < .01, diabetes distress, r_(162)_ = −.26, *p <* .01 and depressive symptoms r_(162)_ = −.31, *p* < .001. This indicated that the more participants’ family members nagged, criticised and argued with them about their diabetes care, the less distressed they felt about their diabetes care and the less depressive symptoms they experienced.

**Table 3:** Correlations of psychosocial variables and diabetes-related clinical variables (n = 162)

Variables	1	2	3	4	5	6	7	8	9	10	11
1. PAID	–										
2. DDS	.79***	–									
3. BDI	.53***	.51***	–								
4. DFBC	−.26**	−.26**	−.31***	–							
5. Diabetes duration	−01	−.04	.06	.05	–						
6. Body Mass Index	.06	.07	.05	−.07	−02	–					
7. HbA1c	.08	.21**	.18*	−.06	−.03	−.03	–				
8. Complications	.10	.09	.25**	−.05	.08	−.001	.15	–			
9. Other conditions	.01	−.03	.05	.07	−.01	.10	−.10	.08	–		
10. Systolic	.16*	.08	.21**	−.02	−.02	.17*	.08	.08	.16*	–	
11. Diastolic	.21**	.01	.11	−.07	−.12	.06	.09	−.11	.07	.63**	–

*p < 0.05 **p < 0.01 ***p < 0.001

*PAID* Problem Area in Diabetes*, DDS* Diabetes Distress Scale

*BDI* Beck Depression Inventory*, DFBC* Diabetes Family Behaviour Scale

*HbA1c* Glycaemic level

### Psychosocial distress and diabetes-related clinical variables

Correlations performed to determine the relationship between psychosocial distress and clinical variables are presented in Table 3. Diabetes distress correlated positively with glycaemic levels (HbA1c), r_(162)_ = .21, *p* < .01, indicating that as diabetes distress increased, blood glucose levels also increased, and vice versa. Depressive symptoms also correlated positively with glycaemic levels r_(162)_ = .18, *p* < .05, diabetes complications, r_(162)_ = .25, *p <* .01 and systolic BP, r_(162)_ = .21, *p* < .01. There was also a significant positive correlation between diabetes-related emotional distress and systolic BP and, r_(162)_ = .16, *p <* .05, and diastolic BP, r_(162)_ = .21, *p <* .01. Thus, as participants’ diabetes-related emotional distress increased, systolic BP and diastolic BP also increased and vice versa. Other clinical variables (BMI, and other medical conditions) did not correlate significantly with any of the psychosocial variables.

### Psychosocial distress and diabetes self-management activities

Results for correlations performed to determine the relationship between psychosocial distress and diabetes self-management activities are presented in Table 4. Diabetes-related emotional distress correlated negatively with only exercise regimen, r_(162)_ = −.22, *p* < .01, indicating that the more participants experienced diabetes-related emotional distress the less they adhered to their exercise regimen, and vice versa. Diabetes distress on the other hand correlated negatively with both dietary regimen (r_(162)_ = −.18, *p <* .05) and exercise regimen (r_(162)_ = −.21, *p <* .01), indicating that the more distressed participants were with their diabetes the less adherent they were to their dietary and exercise regimen and vice versa.

**Table 4:** Correlations of psychosocial variables and diabetes self-management activities (n = 162)

Variables	DSCA Diet	DSCA Exercise	DSCA Medication
1. PAID	−.08	−.22**	−.12
2. DDS	−.18*	−.21**	−.15
3. BDI	−.15	−.15	−.05
4. DFBC	.18*	.25**	.25**

*p < 0.05 **p < 0.01

*PAID* Problem Area in Diabetes*, DDS* Diabetes Distress Scale

*BDI* Beck Depression Inventory, *DFBC* Diabetes Family Behaviour Scale

*HbA1c* Glycaemic level

Depressive symptoms did not correlate with any of the diabetes self-management activities but contrary to the literature, non-supportive family behaviour (i.e. negative family behaviour) correlated positively with all three treatment regimens; diet, r_(162)_ = .18, *p <* .05, exercise, r_(162)_ = .25, *p <* .01, and medication, r_(162)_ = .25, *p* < .01. Thus, the more participant’s family members criticised, nagged and argued with them about their diabetes care, the better they were at maintaining their regimen.

## Discussion

This study investigated how psychosocial distress from diabetes may intercorrelate with each other, and be correlated with clinical variables and diabetes self- management activities.

### Intercorrelations of psychosocial variables

Diabetes-related emotional distress, diabetes distress and depressive symptoms were reciprocally positively associated, corroborating previous findings [28, 29]. This is not surprising as all three variables measure distress and negative emotions in one form or the other. Previous longitudinal evidence suggests that depressive symptoms and diabetes distress are believed to be reciprocally related; depressive symptoms influence diabetes distress, which in turn also influence depressive symptoms [28]. Thus, one condition contributes to the maintenance or worsening of the other condition. Prospective studies also suggest that depression is a risk factor for diabetes (e.g. [30]) while other researches have reported a bidirectional association between depression and type 2 diabetes [31]. However, how much of the depressive symptoms seen in diabetes antedate or results from type 2 diabetes is unclear.

### Psychosocial distress and diabetes-related clinical variables

Of the three psychological variable tested, increased diabetes distress and depressive symptoms were associated with increased gylcaemic levels (and therefore poorer glycaemic control). However, contrary to previous studies [2, 10] there was no relationship between diabetes-related emotional distress and glycaemic levels of participants. Aikens [10] suggests that while depressive symptoms may be of less importance than diabetes-related distress, depressive symptoms may be more important to lifestyle-oriented behaviours and blood glucose self-testing. Perhaps, the fact that participants had to maintain a lifestyle which was not in their normal repertoire, and the challenges of maintaining good glycaemic levels made them feel depressed. Also, it is probable that participants’ distress went beyond living with and managing diabetes (as measured by the PAID) to include more specific distress related to their diabetes care such as frustrations with physicians’ care, emotional burden of having diabetes, lack of support from family and friends and regimen-related distress (as measured by the DDS).

The association of increased diabetes distress with poor glycaemic control corroborates findings by Cummings et al. [5] and Hayashino et al. [6] but is contrary to reports of no association between the variables [32–34]. When people with diabetes are distressed or experience depressive symptoms, their treatment regimen is likely to be affected and this can result in poor self-care and thus poor diabetes control. In the present study, increased levels of depressive symptoms were also associated with more diabetes complications. Perhaps participants worried about their poor glycaemic control, the consequences of their diabetes complications and the possibility of developing more diabetes complications (due to poor glycaemic control), and this may have accounted for the positive association of depressive symptoms with poor glycaemic control and diabetes complications. The present finding suggests that for individuals with high glycaemic levels, it may be worthwhile screening them diabetes distress and depressive symptoms to allow psychological interventions to be used to reduce psychological distress, thereby modifying glycaemic levels.

Increased diabetes-related emotional distress was associated with increased blood pressure levels (both systolic and diastolic), suggesting that when participants were emotionally distressed it adversely influenced their blood pressure levels. In addition, higher levels of depressive symptoms were associated with higher levels of systolic blood pressure. Footman, Roberts, Tumanov & McKee [35] have reported higher levels of psychological distress among people with hypertension compared with a general population, thus, the present finding is not surprising as nearly 80% of participants were hypertensive.

### Psychosocial distress and diabetes self- management activities

The absence of a significant relationship between diabetes-related emotional distress and medication adherence is contrary to reports of lower levels of diabetes-related emotional distress correlating with higher levels of medication adherence [36, 37]. In fact, diabetes distress and depressive symptoms did not also correlate significantly with medication adherence. This suggests that being distressed with diabetes or experiencing depressive symptoms did not determine the extent to which participants adhered to their medication regimen. Perhaps this may be because medication intake was not a challenging regimen to the extent of causing psychological distress, or that any distress experienced did not impede medication intake. However, increased diabetes-related emotional distress and diabetes distress were associated with less adherence to exercise regimen, suggesting that probably, exercise was a more challenging regimen for participants, thus the negative association with psychological distress. As literature suggests, exercise is a treatment regimen that patients constantly struggle to maintain compared with medication intake [38]. Again, higher levels of diabetes distress were associated with poorer dietary adherence but not medication adherence. Zhang et al. [29] have reported that higher levels of diabetes distress is associated with poorer treatment adherence and suggested that psychological intervention is necessary to promote adherence to treatment.

A novel finding in the present study is the significant negative association of non-supportive family behaviour (i.e. negative family behaviour) with diabetes-related emotional distress, diabetes distress and depressive symptoms. This is contrary to our predictions of a positive correlation amongst these variables and previous findings that have reported a strong significant positive association of non-supportive family behaviour with diabetes-related emotional distress [25]. Karlsen and Bru [11] suggested that negative family behaviours such as nagging, criticising and arguing with participants about their diabetes care, results in perceived problems with diabetes and hence increases feelings of diabetes distress. On the contrary, the present findings suggest, the more family members nagged, criticised and argued with participants about their diabetes care, (i.e. what the behaviour checklist labelled as non-supportive behaviour) the less diabetes-related emotional distress, diabetes distress and depressive symptoms participants experienced. In addition, the more what was labelled as negative behaviours were exhibited by family members, the more adherent participants were with their dietary, exercise and medication regimen. This is contrary to reports by Stephens et al. [39] that pressure from partners of older adults with diabetes was associated with decrease in adherence.

A possible explanation is that perhaps participants tried to maintain their treatment regimen just to avoid criticisms, and once that was achieved, they felt less distressed. Thus, in the sample tested, what was labelled as non-supportive behaviours produced positive outcomes for diabetes care. It is also possible that what the “non-supportive” family behaviour checklist measured (nagging, criticising and arguing about diabetes care) was not perceived by participants as non-supportive or negative behaviours. They may have perceived these communications as concerned prodding to improve their wellbeing, especially if such communications were the only concerned attention they were used to. These would then prompt participants to better adhere to their treatment regimen, and feel less distressed. Thus, what Schafer et al. [24] and other researchers [11, 25, 39] labelled as non-supportive behaviours may not have been experienced as such, as these were interpreted by participants as care and concern about their illness, resulting in a positive rather than negative effect on the measured outcome variables. To the best of our knowledge, there is no literature reporting this observation about family support among people with diabetes or other chronic diseases in Ghana or sub-Saharan Africa. This is a matter for further research, to investigate patients’ perceptions and interpretation of communications of what they perceived as positive or negative family support. What communications are presently being used and what communications work best in encouraging patients.

Based on the above findings, the association of increased diabetes distress with poorer glycaemic control and poorer adherence to diet and exercise regimen, suggests the need for healthcare providers to screen patients who may be reporting high levels of distress associated with maintaining their diabetes regimen and offer them support. Psychologists should be routinely part of the multidisciplinary team managing diabetes patients such that patients are properly evaluated and have easy access to appropriate professional help. In addition, screening newly diagnosed patients for depression is warranted, as depression is a risk factor for incident diabetes as well as pre-existing depression being a possible cause of the poor diabetes control. Healthcare providers should offer education to patients to discuss issues of diabetes complications, allay patients’ fears, and assure them that adhering to diabetes regimen and maintaining good glycaemic levels can delay or avert diabetes complications. Patients with poor glycaemic control and diabetes complications should also be screened to detect early symptoms of distress and depression to prevent major depressive disorders.

If criticisms, naggings and arguments from family members were positively related to adherence, then perhaps praises and accolades (supportive/ positive family behaviours) may improve adherence even more. Healthcare providers should involve family members in the care of patients and educate them on the importance of showing concern for patients in encouraging ways other than nagging criticizing and arguing with them.

### Limitations

While this study presents important findings, there are some limitations. First, participants were conveniently selected from only four hospitals in Accra, three of which are government hospitals, and one being a private hospital. Therefore, the study cannot be generalised to all public or private hospitals. Second, since participants were tested during their clinical review, they may have been more focused on seeing their healthcare providers than paying full attention to the study they were involved in and may have responded to some of the questions in haste. Third, some demographic and clinical characteristics of participants may also be a limitation worth noting. Participants tested were mostly women, which was characteristic of the population tested. Glycaemic levels of participants indicated a fairly well controlled sample and most of the participants were on oral medication, which could suggest a lower burden of diabetes. Very few participants were on insulin or a combination of insulin and oral medication but this was also typical of the population tested and not because of the researchers missing participants on insulin who may have poorer outcomes and perhaps a more challenging diabetes care. It must be noted that Ghana has since the late 90s implemented two national diabetes programmes that have trained three-member diabetes teams (a doctor, nurse-diabetes educator and dietitian). On each clinic contact, there is active diabetes education by the diabetes educators and contact with diet therapist if needed. In addition, patients who consistently present with uncontrolled glycaemic levels are referred to the National Diabetes Management and Research Centre for further treatment. These may have contributed to the relatively lower glycaemic levels recorded in the study. Finally, the measure of maintaining a healthy diet was self-reported and not by objective assessment. Thus, it is probable that participants may not have been accurate in their report. Future studies should assess this measure objectively.

In spite of the above limitations, this study provides useful information about psychosocial distress in a sample of people with type 2 diabetes in Ghana.

## Conclusion

This study is the first in Ghana to assess psychosocial distress amongst people with type 2 diabetes. Diabetes distress was positively associated with glycaemic levels, but negatively associated with adherence to dietary regimen and exercise regimen. Depressive symptoms were also positively associated with glycaemic levels and diabetes complications, while there was a negative association between diabetes-related emotional distress and adherence to exercise regimen. Of particular interest is the novel finding on family support. What was labelled as non-supportive family behaviour correlated positively with adherence to treatment regimen, suggesting that perhaps, showing concern to patients in encouraging ways could improve adherence even more. This study has also shown the need for people with type 2 diabetes to be screened for psychosocial distress, especially, that related to their treatment regimen. Psychosocial care should be appropriately incorporated in the care provided to patients with type 2 diabetes in Ghana.

## Data Availability

The datasets generated during the current study are not publicly available because the data forms part of a bigger study from which other manuscripts are being written. Thus, all data and supporting materials will be made available from the corresponding author (on agreement with the co-authors) on reasonable request.
